# The Mechanism of Heat Stress Resistance During Spermatogenesis in Turpan Black Sheep

**DOI:** 10.3389/fvets.2022.846981

**Published:** 2022-06-13

**Authors:** Yukun Song, Xi Zhao, Aikebaier Aihemaiti, Aerman Haire, Yu Gao, Chao Niu, Peng Yang, Guoshi Liu, Gongxue Jia, Abulizi Wusiman

**Affiliations:** ^1^Department of Animal Science, College of Animal Science, Xinjiang Agricultural University, Urumqi, China; ^2^Tuokexun County Huishang Ecological Animal Husbandry Co., Ltd., Turpan, China; ^3^National Engineering Laboratory for Animal Breeding, Beijing Key Laboratory for Animal Genetic Improvement, College of Animal Science and Technology, China Agricultural University, Beijing, China; ^4^Key Laboratory of Adaptation and Evolution of Plateau Biota, Northwest Institute of Plateau Biology, Chinese Academy of Sciences, Xining, China

**Keywords:** heat stress, spermatogenesis, meiosis, Turpan black sheep, RNA-seq

## Abstract

Heat stress can affect the reproductive function of livestock and cause harm to animal production, which can seriously damage the economic interests of livestock producers. Therefore, it is important to explore the effect of heat stress on reproductive function to improve livestock production. In this study, the experimental animals Turpan black sheep and Suffolk sheep were selected as controls, each with 10 sheep, and the reproductive physiological performance was measured in Turpan, China from April to August when there was no heat stress to strong heat stress. The results showed that the sperm density, vitality, and kinematic parameters of Suffolk sheep were significantly lower than that in Turpan black sheep (*p* < 0.01) after heat stress, while the sperm acrosome malfunctions and DNA damage were significantly higher in Suffolk sheep (*p* < 0.01). In addition, the endogenous levels of reproductive hormones and oxidative stress indicators in the blood of Turpan black sheep were stable before and after heat stress treatment, while Suffolk sheep showed different degrees of fluctuations. There was no significant difference in testicular histomorphology between the two after heat stress treatment. However, Suffolk sheep showed a significantly decreased number of spermatocytes after heat stress treatment (*p* < 0.05). It was found that during meiosis, the proportion of cells in the meiotic zygotene stage of Suffolk sheep was significantly higher than that of Turpan black sheep. To investigate the mechanism of normal spermatogenesis in Turpan black sheep under heat stress, we performed RNA-Seq analysis on the testis. The results showed that there were 3,559 differential genes in Turpan black sheep before and after heat stress, with 2,118 up-regulated genes and 1,441 down-regulated genes. The enrichment analysis of GO and KEGG showed that the differential genes are mainly involved in cellular component organization or biogenesis, cell cycle process, mitotic cell cycle process, meiotic cell cycle process, double-strand break repair and Rap1 signaling pathway, Ras signaling pathway, Cell cycle, signaling pathways regulating pluripotency of stem cells Oocyte meiosis. Genes related to spermatogenesis, *SYCP2, TDRD9, BRDT, CEP120, BRCA1*, etc. were significantly up-regulated in Turpan black sheep after heat stress. In summary, our results showed that the up-regulation of genes involved in spermatogenesis protects the normal production of sperm in Turpan black sheep under HS, thereby achieving normal reproductive function.Our research systematically elucidated the mechanism of heat stress resistance during spermatogenesis in Turpan black sheep and provided potential possibilities for the subsequent breeding of new heat-resistant breeds.

## Introduction

Heat stress (HS) is the sum of the body's non-specific physiological responses to a high-temperature environment (1). Almost all animals would suffer from HS in a high-temperature environment (2). In recent years, with the industrialization and intensification of animal husbandry, the research on the stress of livestock and poultry has become a hot topic in the field of animal research (3, 4). HS can cause a series of body's sub-health or functional disorders, mainly including heat wheezing, increased tension of the cardiovascular system, accelerated heart rate, reduced secretion of digestive juices, increased oxidative metabolism, and decreased immunity (5), which results in a decline in production and reproductive performance. Therefore, studying the mechanism of HS resistance is very important to improve the livestock and poultry industry.

Testicular temperature below core body temperature is one of the necessary conditions for sheep to produce normal and highly viable sperm. However, when the testis is exposed to a high-temperature environment, it is extremely prone to induce irreversible damage. Previous studies have illustrated that the abnormal rate of sperm would be increased obviously and the viability of sperm would be decreased after HS (6). Additionally, HS can also induce germ cell apoptosis, DNA damage, and abnormal maturation of sperm (7). This is most likely due to the influence of HS on the concentration of amino acids, fatty acids, minerals, enzymes, antioxidants, growth factors in the spermatogenesis environment, or the activity of enzymes related to spermatogenesis (8). For example, Merino rams exposed to a high temperature of around 35°C for 3 days showed a significant decrease in sperm motility during the following 15 to 35 days (9). In addition, abnormal sperm motility, morphology, and quantity of sperm will also significantly decrease. The pachytene spermatocytes and round sperm cells were considered to be susceptible to HS (10), increasing the apoptosis, thus inhibiting the spermatogenesis. The increase in ambient temperature leads to the production of intracellular reactive oxygen species (ROS) (11), which damage DNA, inhibit cell proliferation, and induce apoptosis (7).

Turpan region belongs to the typical continental warm temperate desert climate, with abundant sunshine, abundant heat, and extremely dry. In summer, the maximum air temperature can reach 49.6°C, the surface temperature can reach more than 70°C. However, in this extreme environment, there is special livestock in the Turpan region – Turpan black sheep. Turpan black sheep is formed under long-term breeding by farmers and herdsmen in Toksun County of the Turpan region. It is one of the excellent local sheep breeds in China and is bred by Bayanbulak sheep, Kazak sheep, Karakul sheep, and other breeds. Turpan black sheep can not only adapt to the natural climatic conditions of cold and windy winter, but also adapt to the arid and hot climate in the Turpan area, and grow fast in the soil with strong lignification and arid saline-alkali. Even under extreme heat condition, Turpan black sheep still produce healthy sperm. However, when Suffolk sheep (12) were brought in Turpan, male Suffolk sheep showed severe reproductive dysfunction during the summer, failing to produce sperm normally. Hence, we thought that Turpan black sheep may have strong resistance to HS. To prove this hypothesis, we compared hormone levels, sperm production, and germ cell development of Turpan black sheep and Suffolk sheep before and after natural HS treatment. Combining the testicular transcriptome analysis, we identified HS signaling pathways and related molecular mechanisms and illustrated the mechanism of Turpan black sheep testicular resistance to HS. These results provide a new direction for sheep breeding to deal with the negative effects of HS on sheep reproduction.

## Materials and Methods

### Ethics Statement

All experiments were performed following the approved guidelines. All procedures were reviewed and approved by the Ethics Committee of Xinjiang Agricultural University (Protocol Permit Number: 2020032, 7 May 2020).

### Animals

The experiment was carried out in Toksun County, Turpan Region, Xinjiang, China from April to August 2021. The ambient temperature (AT) and relative humidity (RH) were continuously measured using a digital thermometer (Xiaomi Technology Co., Ltd.) for 104 days from April 28. Temperature and heat index (THI) was calculated as follow: THI = (1.8^*^AT+32)–(0.55–0.55^*^RH^*^0.01)^*^(1.8^*^AT-26). THI ≤ 72, no HS; 73 ≤ THI ≤ 77, mild HS; 78 ≤ THI ≤ 89, moderate HS; THI ≥ 90, severe HS.

At the beginning of the experiment, 1.5-year-old rams were chosen with healthy body conditions and normal reproductive function. Six Turpan black sheep and six Suffolk sheep were kept under the same conditions of breeding and management. The external or internal body temperature of animals was measured using an infrared digital thermometer or a mercury thermometer.

### Detection of Hormone Levels and Antioxidant Indicators

Blood samples were collected and centrifuged to separate the serum, and stored at−80°C. The radioimmunoassay was used to determine follicle-stimulating hormone (FSH), luteinizing hormone (LH), cortisol (COR), testosterone (T), cholesterol (CHOL), catalase (CAT), superoxide dismutase (SOD), malondialdehyde (MDA), and glutathione peroxidase (GSH-Px).

### Determination of Sperm DNA Fragmentation Index

Sperm chromatin dispersion (SCD) test was carried out on 500 sperm per group, and the ratio of sperm head diameter to halo size: the statistics of large halo sperm, medium halo sperm, small halo sperm, no halo sperm, and degenerative sperm. Sperm DNA fragmentation index (DFI) = (small halo sperm + no halo sperm + degenerated sperm)/500 × 100%.DFI parameter values: (1) DFI < 15%, sperm with good DNA integrity. (2) 15% ≤ DFI ≤ 30%, sperm with average DNA integrity. (3) DFI > 30%, sperm with poor DNA integrity.

### Testis Sample and Immunostaining

Testis fixed in 4% paraformaldehyde (PFA) at 4°C was dehydrated stepwise through an ethanol series and embedded in paraffin. About 5 μm sections were cut using Leica RM2235 Slicing Machine. After dewaxing and rehydration, sections were stained with H&E. For immunostaining, sections were boiled in 10 mM sodium citrate buffer (pH 6.0) for 20 min. On cooling to room temperature, samples were washed in PBS and blocked with 10% donkey serum in PBS for 1 h. Then sections were incubated with primary antibodies diluted in PBS containing 3% bovine serum albumin (BSA) at 4°C overnight and washed in PBS before incubating with secondary antibodies for 2 h at room temperature. After washing in PBS thoroughly, sections were incubated with DAPI and mounted on slides for immunofluorescence analysis in Leica DMR Fluorescence Microscope.

The antibodies used are as follows: anti-UCLH1 (Abcam, USA, Ms mAb; 1:400 dilution, 0.5 μg/ml), anti-SYCP1 (Santa Cruze, USA, Rb pAb; 1:100 dilution, 0.5 μg/ml), anti-SOX9 (Abcam, USA, Rb pAb; 1:400 dilution, 0.5 μg/ml), anti-PNA (Vector Labs, 1:500 dilution), Alexa-Fluor-488 Affinipure Goat Anti-Mouse IgG (Abcam, USA, 1:500, 4 μg/ml), Alexa-Fluor-555- Dnk pAb to Rb IgG (Abcam, USA, 1:500, 4 μg/ml).

### Chromosome Spreading

Refer to the method for meiotic cell plating and make some modifications based on it. Tissue samples frozen in liquid nitrogen were thawed and thawed in a 37°C water bath. The tissue was minced in a centrifuge tube to prepare a single cell suspension, the supernatant was discarded and left for 20 min, and then 100 mM sucrose solution was added. Spread the suspension evenly on the glass slide, place it in a heat and humidity box for more than 2 h, and dry the prepared slides after 30 min. Prepared slides were placed in antibody dilution buffer (ADB) to seal for 1 h, and 50 μl of the primary antibody was added dropwise to the slides, sealed with rubber glue, and incubated a 37°C incubator for 24 h. Then add 50 μl secondary antibody and place in a 37°C incubator for 2 h. After washing with PBS, the specimens were stained with DAPI for 30 min and mounted with glycerol for microscopic examination.

### RNA Extraction, Library Construction, and Sequencing

For each group, tissues from three animals were used for RNA extraction, library construction, and sequencing. Briefly, the total cellular RNA of testis was isolated using the Trizol kit (Promega, USA) following the manufacturer's instructions. RNA quality and concentration were measured by using Agilent 2100 Bio-analyzer (Agilent Technologies, Santa Clara, CA) and by RNase-free agarose gel electrophoresis after removing the genomic DNA. Next, Poly (A) mRNA was isolated using oligo-dT beads (Qiagen). All mRNA was broken into short fragments by adding a fragmentation buffer. First-strand cDNA was generated using random hexamer-primed reverse transcription, followed by the synthesis of the second-strand cDNA using RNase H and DNA polymerase I. The cDNA fragments were purified using a QIA quick PCR extraction kit. These purified fragments were then washed with EB buffer for end reparation poly (A) addition and ligated to sequencing adaptors. Following agarose gel electrophoresis and extraction of cDNA from gels, the cDNA fragments were purified and enriched by PCR to construct the final cDNA library. The cDNA library was sequenced on the Illumina sequencing platform (Illumina HiSeq™ 2500) using the paired-end technology by Gene Denovo Co. (Guangzhou, China). A Perl program was written to select clean reads by removing low-quality sequences (there were more than 50% bases with quality lower than 20 in one sequence), reads with more than 5% N bases (bases unknown), and reads containing adaptor sequences.

### Transcript Assembly and Expression Value Estimation

BosGRu_v2.0 was used for the alignment of RNA sequencing reads. Sequencing reads in FASTQ format were mapped to the reference genome, as well as splice junctions were identified using TopHat (13). Cufflinks package (14) was used for genome-guided transcript assembly and expression abundance estimated. First, Cufflinks was used to reconstruct transcripts based on genome annotation, then the transcripts from each sample were merged by cuffmerge. Novel transcripts were extracted from the result with the threshold of “length ≥200 bp and exon number ≥,” and they were compared with three protein databases to obtain function annotation using blastx (15) with E-value cut-off of 1e-5. The databases contained NCBI non-redundant protein database (Nr) (http://www.ncbi.nlm.nih.gov/), KEGG (http://www.kegg.jp/), and GO (http://geneontology.org/). Next, the novel transcripts were integrated with the existing transcript in genome annotation to construct a new gft file. Finally, cuffquant and cuffnorm were used to estimate transcript expression value as FPKM with parameter library normalization methods: classic-fpkm, library types: fr-unstranded.

### Cluster Analysis

DAVID database was used for functional annotation (https://david.ncifcrf.gov/). The significantly changed gene IDs of the RNA-seq data were converted to the IDs consistent with DAVID. Converted IDs were loaded to DAVID listing KEGG pathways and functional clusters.

### Data and Code Availability

The raw sequence data reported in this paper have been deposited in the Genome Sequence Archive (Genomics, Proteomics & Bioinformatics 2021) in National Genomics Data Center (Nucleic Acids Res 2021), China National Center for Bioinformation / Beijing Institute of Genomics, Chinese Academy of Sciences (GSA: CRA005723) that are publicly accessible at https://ngdc.cncb.ac.cn/gsa.

### Statistical Analysis

SPSS version 22.0 software (SPSS Inc, Chicago, US) was used for the analysis of variance for Student's *t*-test and the values were expressed as mean ± SEM. *p* < 0.05 was regarded as a significant difference.

## Results

### The Effect of High Ambient Temperature on the Body Temperature of Sheep

**Figure 1:**
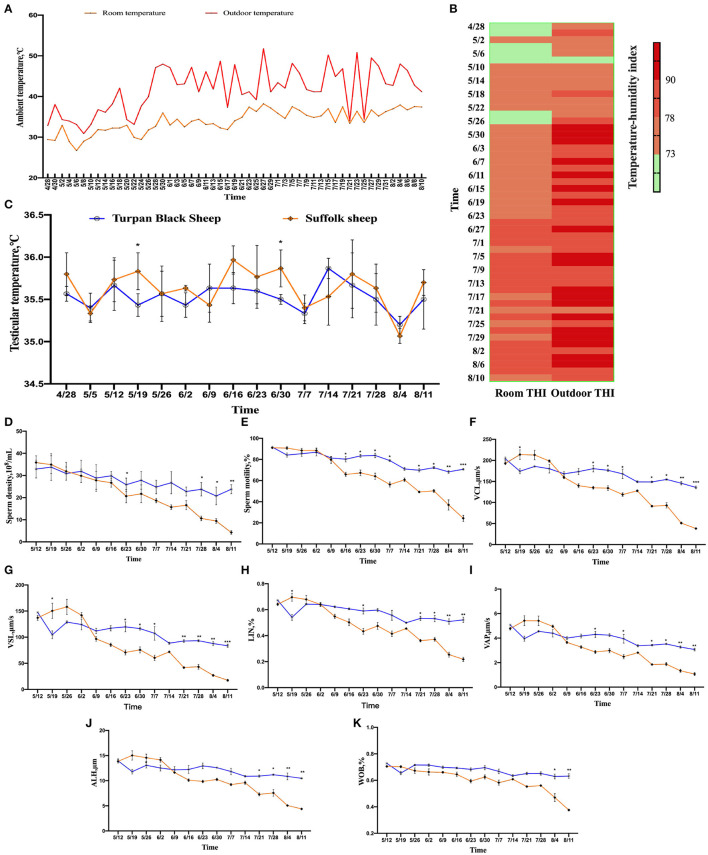
Changes in environmental temperature and THI under heat stress. **(A)** Temperature changes inside and outside the shed from April 28 to August 10. **(B)** Temperature and humidity index change, the darker the color, the more serious the heat stress stimulation. Data were displayed through four measurements per day. **(C)** Testicular temperature changes during heat stress. **(D–K)** Continuous sampling of ram, and sperm motility parameters [**(D)** density, **(E)** vitality, **(F)** Curve line velocity, **(G)** straight line velocity, **(H)** Sperm movement linearity, **(I)** Velocity of the average path, **(J)** Mean side swing amplitude of sperm, **(K)** Sperm wobble].

The results show that after May 26, the temperature increased significantly until the experiment was completed (Figure 1A). THI showed that sheep were under normal or mild HS before May 8. After June 23, the sheep were subjected to intense HS until the experiment was completed (Figure 1B). The temperature of the sheep was measured by infrared thermometers, including the head, ears, abdomen, rectum, and testicles once a week. From May 5th, the temperature of each part of the sheep decreased. With the increase in the ambient temperature, the temperature of the sheep increased significantly. The head temperature of Suffolk sheep was significantly higher than that of Turpan black sheep from June 2 to July 7. The highest temperature of 39.73°C appeared at the same time between them and then decreased to the normal temperature (Supplementary Figure 1A). The temperature of the ear in the two groups varied significantly. In the middle stage of HS, the temperature of Suffolk sheep was always at a high level, which was significantly higher than that of Turpan black sheep, and decreased to the normal temperature after July 28 (Supplementary Figure 1B). There was no significant difference in abdominal temperature between the two groups in the early stage of HS. After June 2, the temperature of Turpan black sheep was stable, while the abdominal temperature of Suffolk sheep was above 38.5°C, and began to decrease on July 21 (Supplementary Figure 1C). The rectal temperature of Suffolk sheep was significantly higher than that of Turpan black sheep before heat stress (*p* < 0.05). With the increase in HS intensity, the rectal temperature of sheep in both groups began to increase, and after June 2, the rectal temperature was in the range of 39–40.5°C (Supplementary Figure 1D). The testicle's temperature is lower than other parts of the body, which is conducive to sperm production. The temperature of the two groups increased after May 5. During heat stress, there was no significant difference in testicular temperature between the two groups (*p* > 0.05) (Figure 1C). Through continuous monitoring of environmental conditions, we found that sheep are under heat stress and that their body has changed.

### HS Alters Sheep Sperm Quality

From May 26, the sperm density of both sheep showed a decreasing trend, and the decrease of Suffolk sheep was more significant than Turpan black sheep (*p* < 0.05). On August 11, the sperm density of Suffolk sheep reached the lowest value of 4.29 × 10^8^/ml, which was significantly lower than that of Turpan black sheep (*p* < 0.01) (Figure 1D).

Meanwhile, the sperm motility of Suffolk sheep decreased significantly (*p* < 0.05) and was significantly lower than that of Turpan black sheep on August 4 and August 11, respectively (*p* < 0.01) (Figure 1E). Subsequently, the values of VCL, VSL, LIN, VAP, ALH, and WOB during HS were analyzed. It was found that the values of Suffolk sheep were significantly lower than those of Turpan black sheep (*p* < 0.05) (Figures 1F–K). These results suggest that HS adversely affects sperm production in Suffolk sheep. After HS, we also found that the semen of Suffolk sheep showed solid form, while the semen of Turpan black sheep showed normal liquid form (**Figure 4C**).

### Changes in Serum Hormone and Antioxidant Indexes in Animals After Heat Stress

**Figure 2:**
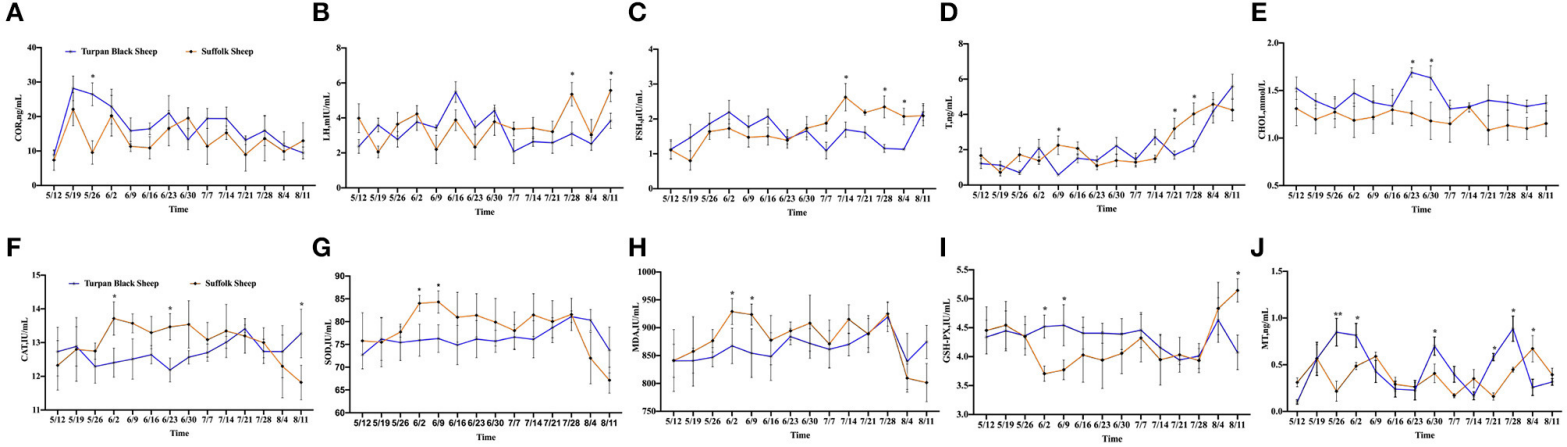
Changes of reproductive hormones and antioxidant indices during HS. **(A)** Hydrocortisone. **(B)** Luteinizing hormone. **(C)** Follicle-stimulating hormone. **(D)** Testosterone. **(E)** Total cholesterol. **(F)** Catalase. **(G)** Superoxide dismutase. **(H)** Malonaldehyde. **(I)** Glutathione synthetase. **(J)** Melatonin. Significance was calculated by *T*-test, *means *P* < 0.05, ** means *P* < 0.01.

After HS, COR, a hormone that responds to stress, was increased in both groups of sheep. Between May 19 and May 26, the COR value in Suffolk sheep decreased significantly (*p* < 0.05), and then gradually returned to the initial level in both groups (Figure 2A). The concentrations of LH and FSH were determined. Before June 30, there were no significant differences in serum LH and FSH concentrations between the two groups (*p* > 0.05). The LH concentration of Turpan black sheep decreased on June 30 and was significantly lower than that of Suffolk sheep on July 28 and August 11 (*p* < 0.05) (Figure 2B). From July 14 to August 4, the FSH content of Turpan black sheep was significantly lower than that of Suffolk sheep (*p* < 0.05) (Figure 2C). T levels did not change significantly from May 12 to July 14. After July 14, the T levels in both groups gradually increased as the temperature increased. On July 21 and 28, the T content of Turpan black sheep was significantly lower than that of Suffolk sheep, and then reached the highest of 5.59 ng/ml on August 11 (Figure 2D). Interestingly, we found that the concentration of total cholesterol of Turpan black sheep during HS treatment was higher than that of Suffolk sheep. From June 23 to 30, the total cholesterol content of Turpan black sheep was significantly higher than that of Suffolk sheep (*p* < 0.05), and then the concentration of the two groups returned to the normal level (Figure 2E). Then, the levels of CAT, SOD, MDA, and GSH-Px were determined. With the increase in THI index, the concentrations of CAT, SOD, and MDA of Suffolk sheep gradually increased, and the concentrations of CAT, SOD, and MDA of Suffolk sheep were significantly higher than those of Turpan Black sheep from June 2 to 9 (*p* < 0.05). However, the changes in Turpan black sheep were not obvious (Figures 2F–H). After May 26, the GSH-Px change trend of Turpan black sheep was stable, while Suffolk sheep decreased. From June 2 to June 9, the content of GSH-Px of Turpan black sheep was significantly higher than that of Suffolk sheep (*p* < 0.05). On August 11, the content of GSH-Px in Turpan black sheep was significantly lower than that in Suffolk sheep (*p* < 0.05) (Figure 2I). It was found that during HS, MT of Turpan black sheep changed significantly, and peaked on May 26, June 2, June 30, and July 28, respectively, which was significantly higher than that of Suffolk sheep (*p* < 0.05) (Figure 2J). Our results show changes in reproductive hormones and antioxidants in sheep before and after HS.

### Changes in Sperm DFI and Acrosome Morphology After HS

**Figure 3:**
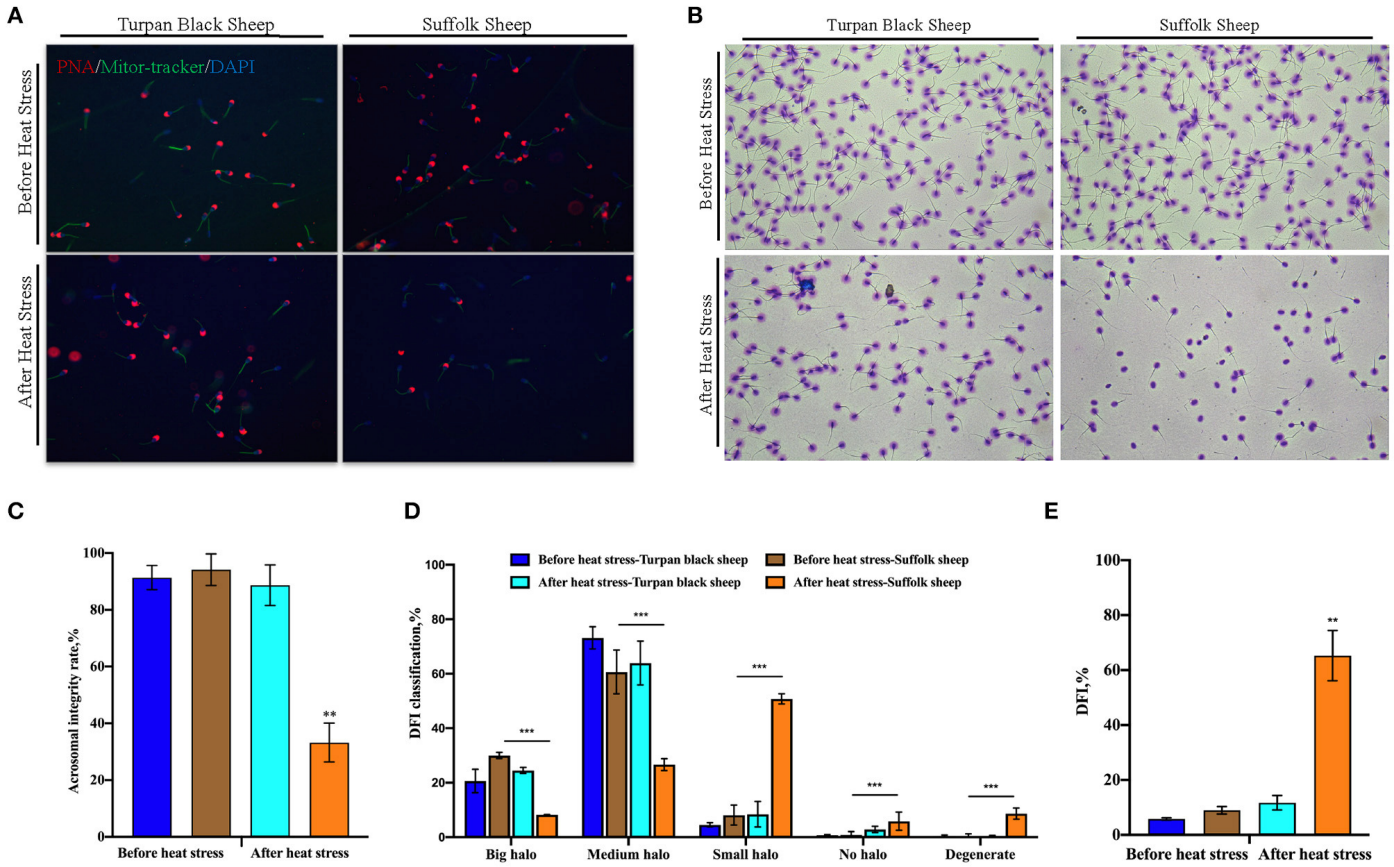
Changes of sperm acrosome morphology and DNA damage after HS **(A)** 200 × confocal laser microscopy: PNA (red fluorescence) labeled sperm acrosome; Mitor Tracker labeled mitochondria; DAPI (blue fluorescence) marks the nucleus. **(B)** SCD sperm DFI images. **(C)** Sperm acrosome morphological statistics. **(D)** SCD results showed the percentage of large halo, medium halo, small halo, non-halo and degenerate sperm. **(E)** DFI statistics. Significance was calculated by *T*-test, ** means *p* < 0.01, *** means *p* < 0.001.

PNA staining showed that there was no significant difference in acrosome morphology of Turpan black sheep, while Suffolk sheep showed a very significant decrease after HS (*p* < 0.01) (Figures 3A,C). On the other hand, DFI staining showed that the proportion of degraded sperm in Suffolk sheep was extremely significantly increased after HS (*p* < 0.01), the DFI index was 65.2%, which was extremely significantly increased, and sperm DNA was severely damaged (Figures 3B,D,E). Statistical results show severe sperm damage in Suffolk sheep after heat stress.

### Morphological Change of Testis

**Figure 4:**
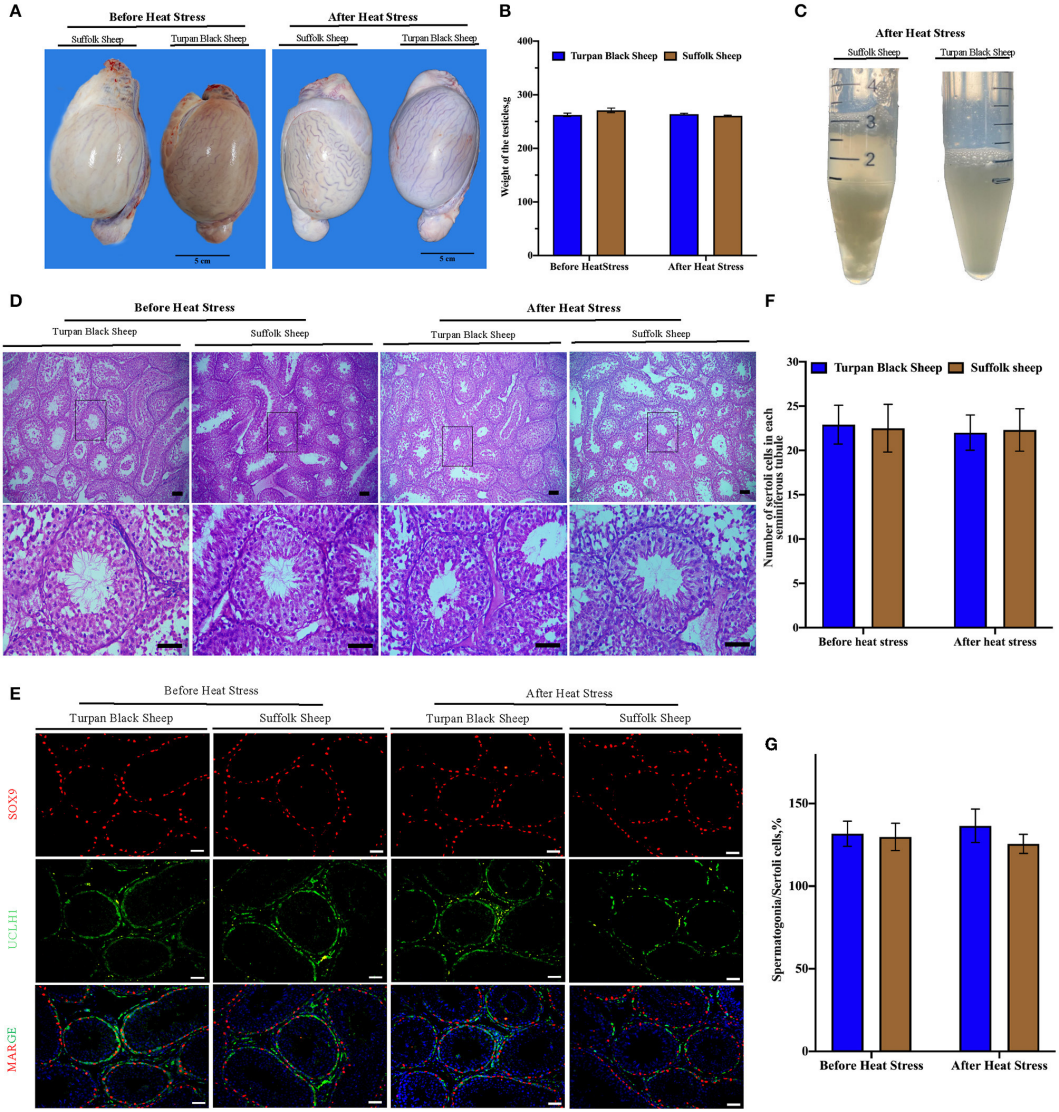
Morphological characteristics of testis and semen after HS. **(A,B)** Typical testicular images and weight comparison after HS. **(C)** Semen morphology after HS. **(D)** Morphological analysis after H&E staining showed that spermatogonial cells, Sertoli cells, spermatocytes, and spermatozoa were arranged orderly in the convoluted tubules of the testis. **(E)** SOX9 (red fluorescence)-labeled Sertoli cells and UCLH1 (green fluorescence)-labeled spermatogonial cells. **(F)** Quantitative analysis of Sertoli cells in spermatogenic tubules. **(G)** The proportion of spermatogonia in 500 Sertoli cells in spermatogenic tubules. Scale = 50 μm, significance was calculated by *T*-test.

The sheep were anesthetized and the testes and epididymis were removed. There was no significant difference in testicular weight between Turpan black sheep and Suffolk sheep before and after HS (*p* < 0.05) (Figures 4A,B). The results of H&E staining of testis showed that testis morphology was intact, and all types of germ cells including spermatogonial cells, spermatogamous cells, round sperm, and long sperm developed normally without significant differences (Figure 4D).

### Immunofluorescence Analysis of Sertoli Cells and Spermatogonial Cells in Testis After HS

Next, the testis germ cells were analyzed. Immunofluorescence staining was performed on sheep testis before and after HS treatment using the antibody SOX9 of supporting cells (Figure 4E). After HS, there was no significant difference in the number of positive cells of Sertoli cells in each spermatogenic tubule between Turpan black sheep and Suffolk sheep, indicating that HS did not affect the number of Sertoli cells (Figure 4F). Testis was then immunofluorescent stained with spermatogonial antibodies UCLH1 and SOX9. The proportion of spermatogonial cells in 500 Sertoli cells was counted to analyze whether spermatogonial cells were reduced in spermatogenic tubules (Figure 4E). There were no significant differences in spermatogonial cells between the two groups (Figure 4G), indicating that HS did not affect the development of Sertoli cells and spermatogonial cells.

### Meiotic Progression Is Blocked in Suffolk Sheep After HS

**Figure 5:**
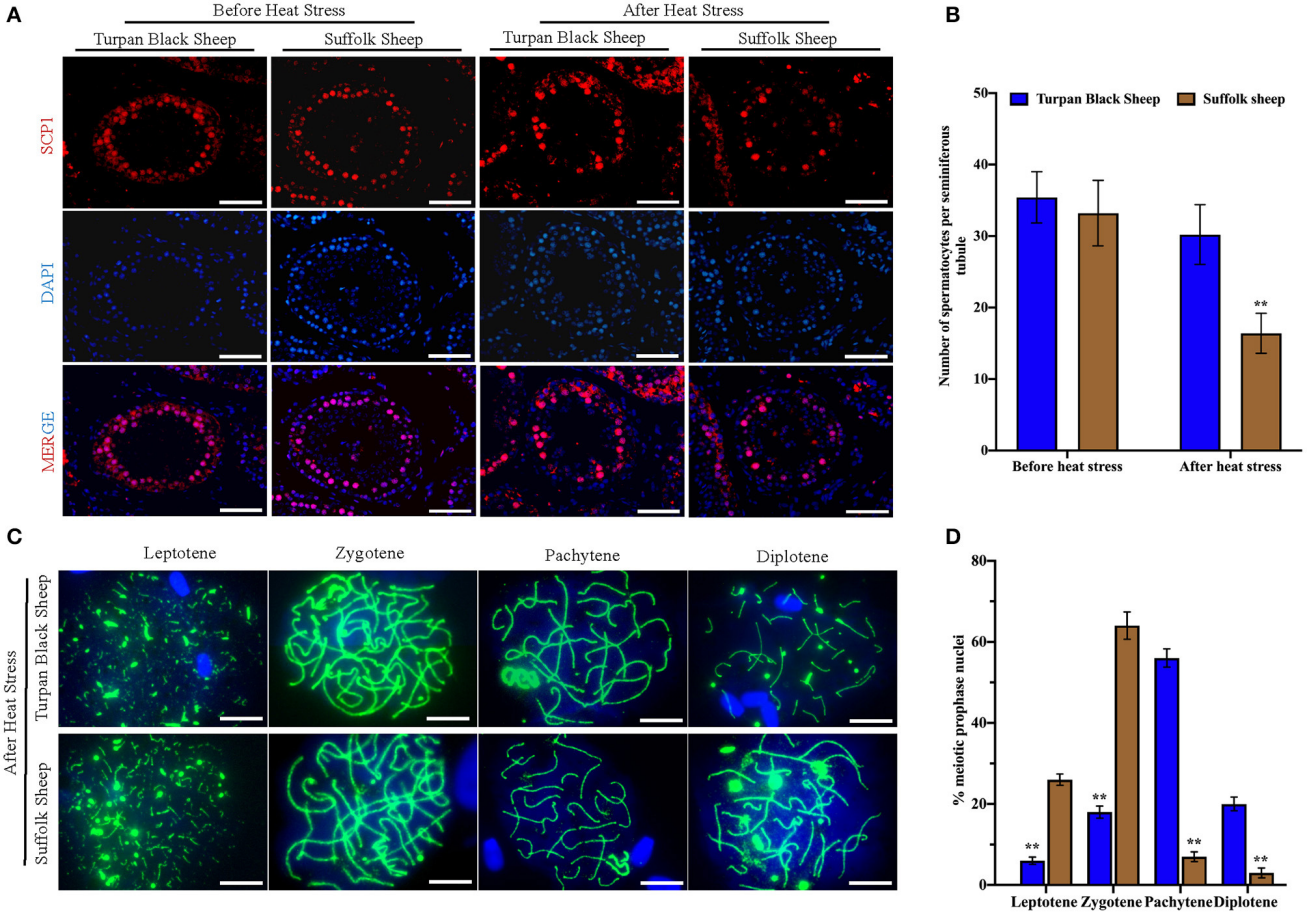
Comparison of spermatocytes and meiosis slices after HS. **(A)** Spermatocyte labeled with SCP1 (red fluorescence) and nucleus labeled with DAPI (blue fluorescence), Scale = 50 μm. **(B)** Number of spermatocyte positive cells in spermatogenic tubules. **(C)** The process of meiosis was analyzed by the method of meiotic splicing and the synaptic complex is labeled by SYCP1 (green fluorescence), Scale = 5 μm. **(D)** The proportion of cells at different stages of meiosis. Significance was calculated by *T*-test, ** means *p* < 0.01.

To find out the cause of reduced sperm count, immunofluorescence staining was performed with spermatocyte antibody SCP1, and spermatocyte statistics were performed (Figure 5A). The spermatocyte number in spermatogenic tubules of Suffolk sheep testis was significantly decreased (16.4 ± 2.8 vs. 33.2 ± 4.6) after HS (*p* < 0.01). However, there was no significant change in spermatocyte number (35.4 ± 3.6 vs. 30.2 ± 4.2) in spermatogenesis tubules of Turpan black sheep (Figure 5B). The results showed that HS had no significant effect on spermatocyte number in the testis of Turpan black sheep, but had a significant effect on Suffolk sheep. To determine the period of meiosis interruption in Suffolk sheep, we analyzed the entire meiotic phase I using meiotic plates. SYCP1 antibody staining was performed on the cells of linea, diplotene, pachytene, and diplotene of Turpan black sheep and Suffolk sheep after HS (Figure 5C). Both groups of sheep could complete the first meiosis. However, the cell proportion of Suffolk sheep was higher than that of Turpan black sheep (64.1 ± 3.4 vs. 18.0% ± 1.5%) (Figure 5D). Therefore, it was speculated that not all spermatocytes of Suffolk sheep could complete meiosis under HS, and a large number of spermatocytes were blocked in the zygotene and pachytene, followed by apoptosis. SOX9 and PNA reagents were used to stain sperm in spermatogenic tubules, and the number of spermatogenic tubules in 500 Sertoli cells was counted (Supplementary Figure 2). The results showed that the number of sperm in the spermatogenesis tubule of Suffolk sheep was significantly decreased after HS compared with that of Turpan black sheep (1,081.2 ± 103.1 vs. 2,642.3 ± 86.2) (Figure 3B). In conclusion, the reduction of spermatocytes and meiosis arrest ultimately lead to a decrease in sperm production.

### Transcriptome Characteristics of Sheep Testis Before and After HS Treatment

**Figure 6:**
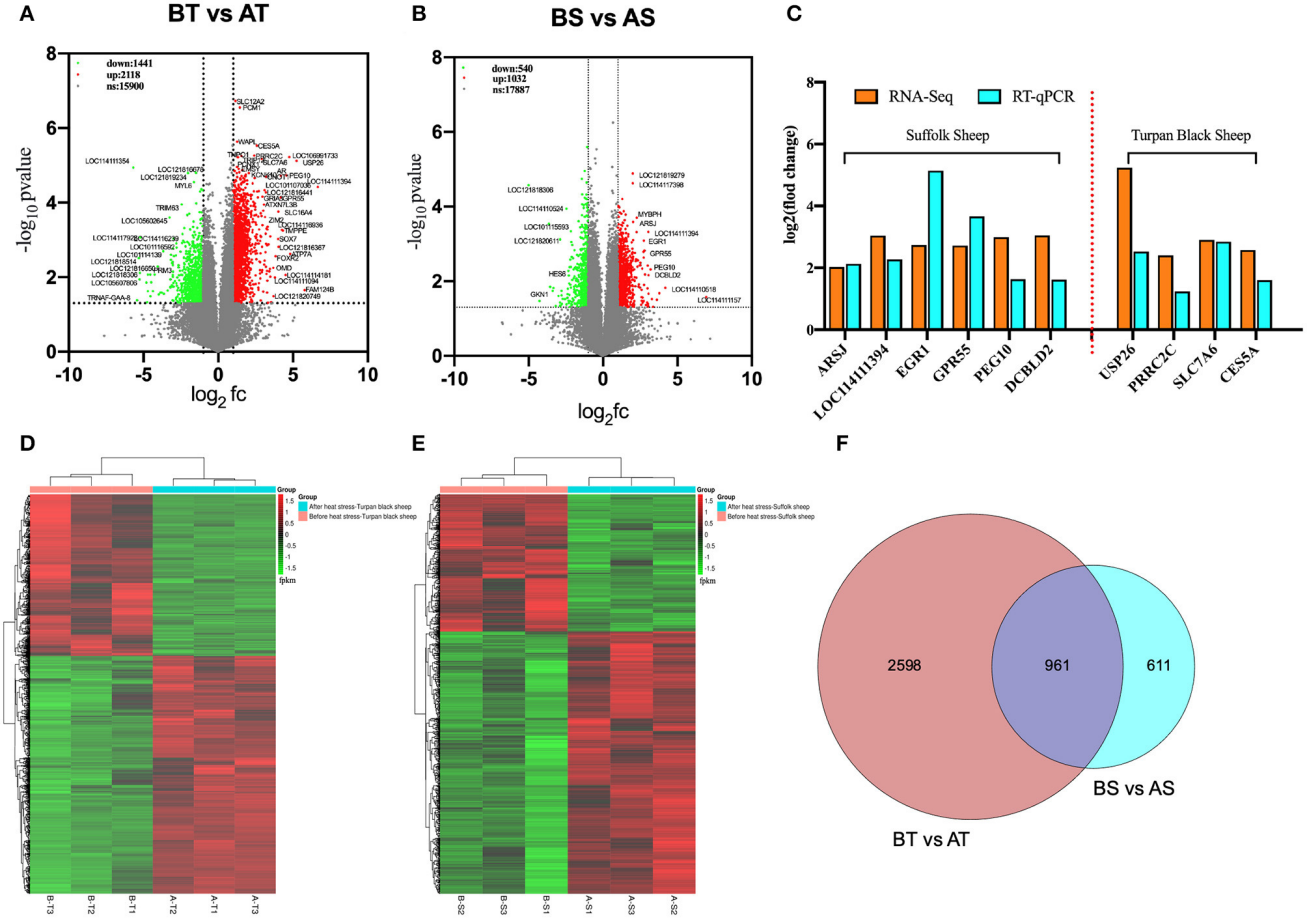
Cluster analysis of differentially expressed genes in the testis transcriptome of Turpan black sheep before and after HS treatment. **(A,D)** Volcano map and heat map of testicular transcriptome differential genes in Turpan black sheep before and after HS. **(B–E)** Volcano plots and heat maps of differential genes in the testis transcriptome of Suffolk sheep before and after HS. **(C)** The trends of differentially expressed genes in RNA-seq and q-PCR. **(F)** Venn diagram representing the common differentially expressed gene distribution. In the volcano plot, red represents up-regulated genes, green represents down-regulated genes, and gray represents genes with no significant difference. The volcano plot showed that the differentially expressed genes satisfy the correction condition *p* < 0.05.

Transcriptome sequencing of 12 representative testes was performed through a detailed description of testicular histology before and after HS treatment to determine which genes were altered during specific stages of spermatogenesis. We obtained 71333670, 76629072, 134221432, and 125842522 clean reads from transcriptome sequencing of testis tissues of Turpan black sheep and Suffolk sheep before and after HS treatment. A total of 95.14, 95.12, 94.64, and 95.00% clean reads were compared to the sheep genome. The overall gene expression analysis showed that there were 390 differentially expressed genes in Turpan black sheep compared to Suffolk sheep before HS, containing 200 up-regulated and 190 down-regulated genes (Supplementary Figure 3A). After HS, there were 311 differentially expressed genes in Turpan black sheep compared to Suffolk sheep, containing 211 up-regulated and 100 down-regulated genes (Supplementary Figure 3G). There were 1,562 differentially expressed genes before and after HS compared to Suffolk sheep, containing 1,032 up-regulated and 540 down-regulated genes (Figure 6B). There were 3,559 differentially expressed genes in Turpan black sheep and Suffolk sheep before and after HS, with 2,118 up-regulated and 1,441 down-regulated (Figure 6A). The differentially expressed genes among the groups were analyzed by clustering, and those clustered together where those with similar expression patterns. The differentially expressed genes between the groups were clustered, and the genes with similar expression patterns were clustered together. The colors in the heat map are compared horizontally. It can be seen from the heat map that all the differential gene sets of the repeat groups are clustered together, indicating that the same sheep gene expression pattern is the same under the same conditions, which is consistent with the heat map of the cluster analysis (Figures 6D,E) (Supplementary Figures 3B,H).

We randomly selected 10 differentially expressed genes in RNA-Seq for verification. After the analysis of RT-qPCR experimental data, it was consistent with the high and low gene expression trends obtained by the RNA-seq transcript sequencing results (Figure 6C), which shows that the transcriptome sequencing data is more reliable, and the differentially expressed genes between different groups can be more accurately detected and selected.

### Effect of HS Treatment on the Gene Expression Pattern of Sheep

Subsequently, GO enrichment and KEGG pathway analysis were performed on the differential genes of Turpan black sheep and Suffolk sheep before HS treatment. The genes with significant BP enrichment are immune response, regulation of the multi-organism process, regulation of defense response, cellular response to lipid, gland development, regulation of smooth muscle cell proliferation, etc. In CC, genes with significant enrichment are plasma membrane, extracellular exosome, receptor complex, synaptic membrane; MF includes receptor activity, heparin-binding, glutathione transferase activity, protein-lipid complex binding, etc. KEGG was enriched in Malaria, Metabolism of xenobiotics by cytochrome P450, *Staphylococcus aureus* infection, Cell adhesion molecules (CAMs), and Complement and coagulation cascades (Supplementary Figures 3C–F).

**Figure 7:**
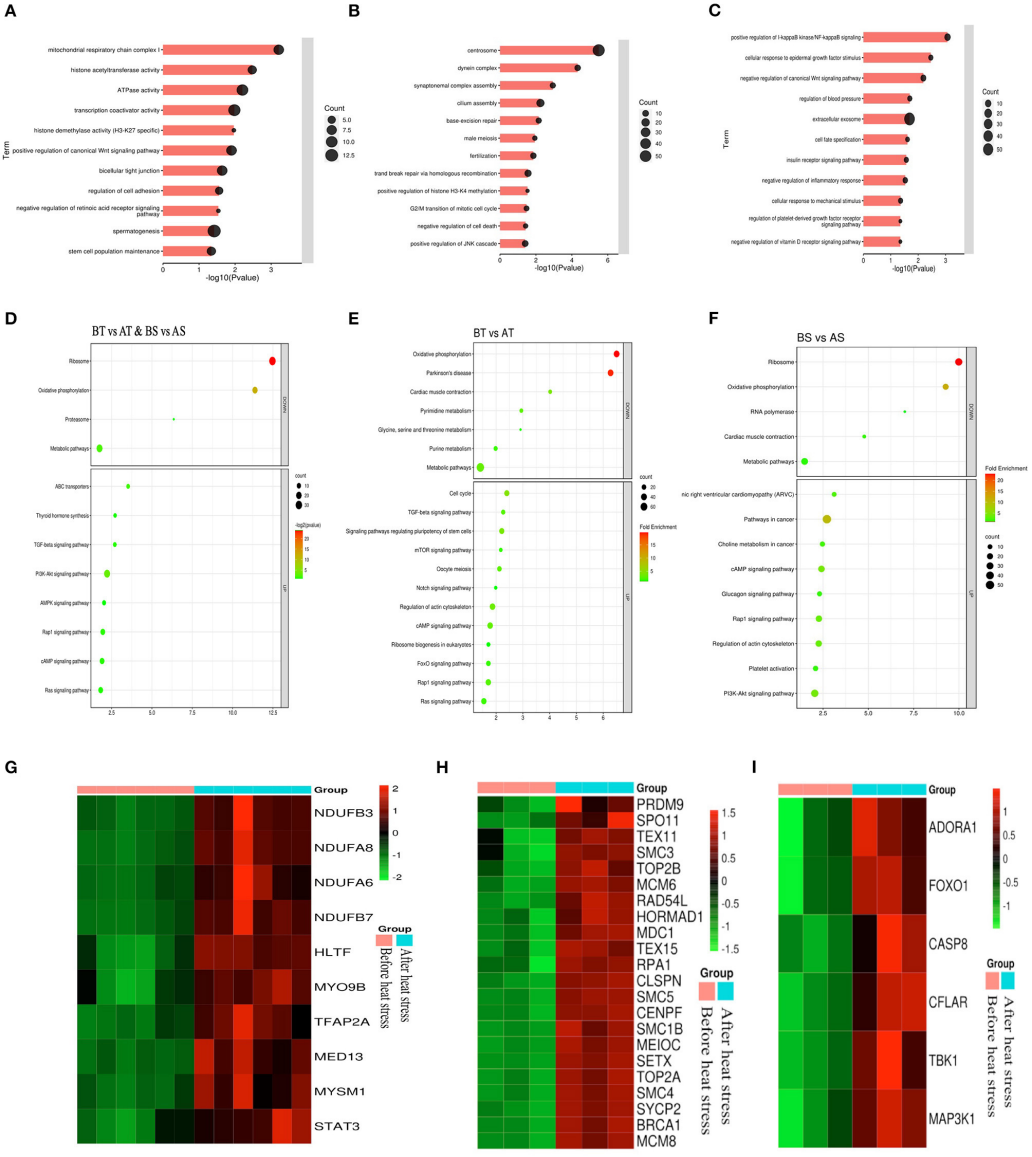
Analysis of common differential genes in the testis transcriptomes of Turpan black sheep and Suffolk sheep before and after HS treatment. **(A–C)** Common differential genes, GO enrichment analysis of Turpan black sheep and Suffolk sheep. **(D–F)** KEGG pathway enriched for differential genes. **(G–I)** Representative differential gene heatmaps.

Next, we analyzed the differential genes of the two sheep before and after HS treatment and found that there were 961 differential genes in common (Figure 6F). First, GO enrichment and KEGG pathway analysis were performed on the differential genes of Turpan black sheep before and after HS treatment. The genes with significant GO enrichment are centrosome, dynein complex, synaptonemal complex assembly, cilium assembly, base-excision repair, male meiosis, fertilization, double-strand break repair via homologous recombination, positive regulation of histone H3-K4 methylation, G2/M transition of the mitotic cell cycle, negative regulation of cell death, positive regulation of JNK cascade, etc. (Figure 7B). KEGG is enriched in PI3K-Akt signaling pathway, cAMP signaling pathway, Rap1 signaling pathway, Ras signaling pathway, Cell cycle, Signaling pathways regulating pluripotency of stem cells, FoxO signaling pathway, mTOR signaling pathway, Notch signaling pathway, etc. (Figure 7E). Then, GO enrichment of differential genes in Suffolk sheep before and after HS treatment significantly showed: positive regulation of I-kappaB kinase/NF-kappaB signaling, cellular (Figures 7C,F). Then, we carried out GO enrichment for the common differential genes of Turpan black sheep and Suffolk sheep before and after HS treatment. The significant pathways are mitochondrial respiratory chain complex I, histone acetyltransferase activity, ATPase activity, transcription coactivator activity, histone demethylase activity (H3-K27 specific), positive regulation of canonical Wnt signaling pathway, bicellular tight junction, regulation of cell adhesion, negative regulation of retinoic acid receptor signaling pathway, spermatogenesis, the stem cell population maintenance, etc. (Figure 7A). The KEGG pathway enrichment includes PI3K-Akt signaling pathway, Rap1 signaling pathway, cAMP signaling pathway, Ras signaling pathway, AMPK signaling pathway, ABC transporters, TGF-beta signaling pathway, Thyroid hormone synthesis, Ribosome, Oxidative phosphorylation, Metabolic pathways, Proteasome Wait (Figure 7D). By comparing the GO enrichment of differential genes and the KEGG pathway, it is found that the pathways between groups are mainly divided into three categories: (1) In Turpan black sheep, it is related to the regulation of spermatogenesis and meiosis; (2) In Suffolk sheep, it is cell Apoptosis related to inflammatory response and adaptability; (3) Among the shared differential genes are related to oxidative stress adaptive capacity. At the same time, after we selected the landmark genes, we found that they were all up-regulated, such as meiosis-related genes *SYCP2, BRCA1, TEX15, SMC4*, S*MC3, SETX, RAD54L, MEIOC, MCM8, MCM6, CENPF, TEX11, SMC5, RPA1*, etc. (Figure 7H), apoptosis-related genes *CASP8, TBK1, CFLAR, FOXO1, ADORA1, CASP8, MAP3K1*, etc. (Figure 7I), oxidative stress-related genes *NDUFB3, NDUFA8, NDUFA6, NDUFB7, HLTF, MYO9B, TFAP2A, MED13, MYSM1, STAT3*, etc. (Figure 7G).

## Discussion

High temperature is an important environmental stress factor that affects livestock production, reproduction, and welfare (16). HS occurs when the body temperature of livestock exceeds the thermoneutral zone, resulting in more heat production than heat loss. Under HS conditions, the heat exchange between livestock and the environment is likely to be blocked, which in turn leads to physical dysfunction in the body (17). Sheep are warm-blooded animals. To ensure their health, survival, and reproduction needs, their body temperature is usually kept within a narrow physiological range (18). The skin is an important way of heat exchange between the environment and the surface of the body. The temperature of the skin is the result of the adjustment of the blood flow of the skin, and the blood flow stops with the adjustment of the heat between the core of the body and the skin (19). It is observed that the temperature of the sheep's skin surface is the highest in summer and the lowest in winter (20). Under HS, the average skin surface temperature of cattle will increase (21). This is similar to the results of this study. We counted the sheep's body temperature during the period from no HS to strong HS and found that the temperature of different parts of the two groups of sheep began to rise when the HS started and then remained at high fever. level, especially in Suffolk sheep (22).

In this study, we found significantly enhanced resistance to male reproductive damage in Turpan black sheep than in Suffolk sheep under HS treatment. Through semen quality testing, we found that there was no significant difference between Turpan black sheep before and after HS, but Suffolk sheep after HS showed the amount of incomplete acrosome and a high proportion of DNA fragmentation. Sperm from mice treated at 35°C for 24 h showed significantly increased mitochondrial ROS production and oxidative DNA damage (23). Similarly, human sperm incubated at 42°C for 3 h can induce negative results in sperm motility, oxidative stress parameters, DNA damage, and cell apoptosis. In cattle, studies have shown that HS can increase the secretion of ACTH and cortisol (24), inhibit the secretion of GnRH and LH, and affect the hypothalamus-pituitary-ovarian axis (25). However, there is no significant difference in Turpan black sheep from the start to the end of HS treatment, although Turpan black sheep showed relatively higher levels of FSH, LH, and T than Suffolk sheep in the late of HS.

Sexually reproducing organisms use meiosis to produce haploid gametes and deliver their genomes to the next generation (26). Our results showed that the number of spermatogonia and Sertoli cells in the seminiferous tubules of Turpan black sheep and Suffolk sheep did not decrease before and after HS, while the spermatocytes of Suffolk sheep showed a significant decrease after HS. Although both groups of sheep could complete the first meiotic division, the proportion of cells in the meiotic zygotene of Suffolk sheep was significantly higher than that of Turpan black sheep. Studies showed that spermatogenesis is particularly sensitive to small temperature fluctuations, and spermatocytes must develop within very narrow isotherms (23, 27–29). Failure of testis thermoregulation and prolonged exposure to high temperatures can lead to male sterility (30). Meiotic prophase spermatocytes and postmeiotic sperm are particularly vulnerable to the effect of HS (10, 31). Furthermore, dysregulation of DNA damage and repair pathways during meiosis has been suggested as a potential mechanism between HS and spermatocyte DNA damage (32). Therefore, we speculate that a large number of cells are blocked in the zygotene and pachytene, followed by apoptosis in SS.

Transcriptome analysis provided more information about the mechanism of HS resistance in the testis of TBS. Our data found some enriched pathways involved in apoptosis, immune response, and oxidative stress. The main enriched pathways are Rap1, AMPK, TGF-β, etc. Rap1 is regulated by a wide range of external stimuli as a molecular switch when stress occurs (33). MAPK is one of the main signaling pathways in mammals and can also be activated by external stimuli, but the activity of the MAPK signaling pathway is stimulated by Rap1 in multiple cells (34, 35). Particularly, the pathways identified in the differential gene enrichment analysis of Turpan black sheep before and after HS are PI3K-Akt, Mtor, Notch, etc. Studies showed that the PI3K-Akt-mTOR signaling pathway can regulate cell growth, proliferation, differentiation, and survival (36), and can regulate the expression of p70S6K and 4EBP1 in testis tissue to regulate spermatogonia proliferation (37). mTOR signaling activates protein translation, participates in the regulation of protein synthesis and energy metabolism, and it is a central regulator of cell proliferation, differentiation, and growth (38). Therefore, we believe that the Turpan black sheep up-regulated meiosis-related genes when they were subjected to HS, causing a cascade of multiple pathways to maintain normal spermatogenesis, thereby reducing the harm caused by HS.

Our study reveals that the hormonal regulation changes and spermatogonia transition, meiotic cell, and haploid germ cell development in Turpan black sheep under HS are regulated by a large number of genetic changes. Transcriptome analysis plays an important role in the screening of candidate genes provided by spermatocytes and spermatogenesis. The up-regulation of related genes involved in spermatogenesis protected the normal production of sperm in the Turpan black sheep after HS treatment further proving that Turpan black sheep have evolved an ability to resist HS in a long-term HS environment.

## Data Availability Statement

The datasets presented in this study can be found in online repositories. The names of the repository/repositories and accession number(s) can be found in the article/Supplementary Material.

## Ethics Statement

The animal study was reviewed and approved by The Ethics Committee of Xinjiang Agricultural University (Protocol Permit Number: 2020032, 7 May 2020).

## Author Contributions

YS: conceptualization, investigation, writing-original draft, and preparation. XZ: conceptualization, data curation, writing-original draft, and preparation. AA: validation, formal analysis, and visualization. AH, YG, and AD: validation. PY and GL: writing-review and editing. GJ and AW: project administration, funding acquisition, and conceptualization. All authors listed have made a substantial, direct, and intellectual contribution to the work and approved it for publication.

## Funding

This work was granted by National Key Research and Development Project (2021YFD1200400) and Strategic Priority Research Program of CAS (XDA26040302). GJ was supported by Qinghai Kunlun Talents programs and Youth Innovation Promotion Association CAS.

## Conflict of Interest

PY was employed by the company Tuokexun County Huishang Ecological Animal Husbandry Co., Ltd. The remaining authors declare that the research was conducted in the absence of any commercial or financial relationships that could be construed as a potential conflict of interest.

## Publisher's Note

All claims expressed in this article are solely those of the authors and do not necessarily represent those of their affiliated organizations, or those of the publisher, the editors and the reviewers. Any product that may be evaluated in this article, or claim that may be made by its manufacturer, is not guaranteed or endorsed by the publisher.

## Supplementary Material

Supplementary Figure 1 Changes in temperature in different parts of the body during heat stress. **(A)** Head, **(B)** ear, **(C)** abdomen, and **(D)** rectum.

Supplementary Figure 2 Analysis of spermatozoa number in spermatogenic tubules after HS. **(A)** SOX9 (green fluorescence) labeled supporting cells, PNA (red fluorescence) labeled sperm, and DAPI (blue fluorescence) labeled nucleus. **(B)** Spermatozoa in 500 sertoli cells. Scale = 50 μm, significance was calculated by *T*-test, ^**^ means *P* < 0.01.

Supplementary Figure 3 Testis transcriptional profile characteristics and differential gene clustering analysis. **(A,B)** Volcano and heat map of testis transcriptome differential genes in Turpan black sheep and Suffolk sheep before HS. **(C–F)** KEGG pathway and cluster analysis of the biological process (BP), cellular component (CC), and molecular function (MF) of differential gene enrichment in Turpan black sheep and Suffolk sheep before HS. **(G,H)** Volcano and heat maps of differentially gene genes in the testicular transcriptomes of Turpan black sheep and Suffolk sheep after HS. **(I)** Cluster analysis of differential genes BP, CC, and MF in Turpan black sheep and Suffolk sheep after HS. In the volcano plot, red represents up-regulated genes, green represents down-regulated genes, and gray represents genes with no significant difference. The volcano plot showed that the differentially expressed genes satisfy the correction condition *P* < 0.05.
