# Supplementary figures and images for: The Mechanism of Heat Stress Resistance During Spermatogenesis in Turpan Black Sheep
Source: Front Vet Sci. 2022 Jun 13;9:846981. doi: 10.3389/fvets.2022.846981 (PMC9236572; doi:10.3389/fvets.2022.846981)

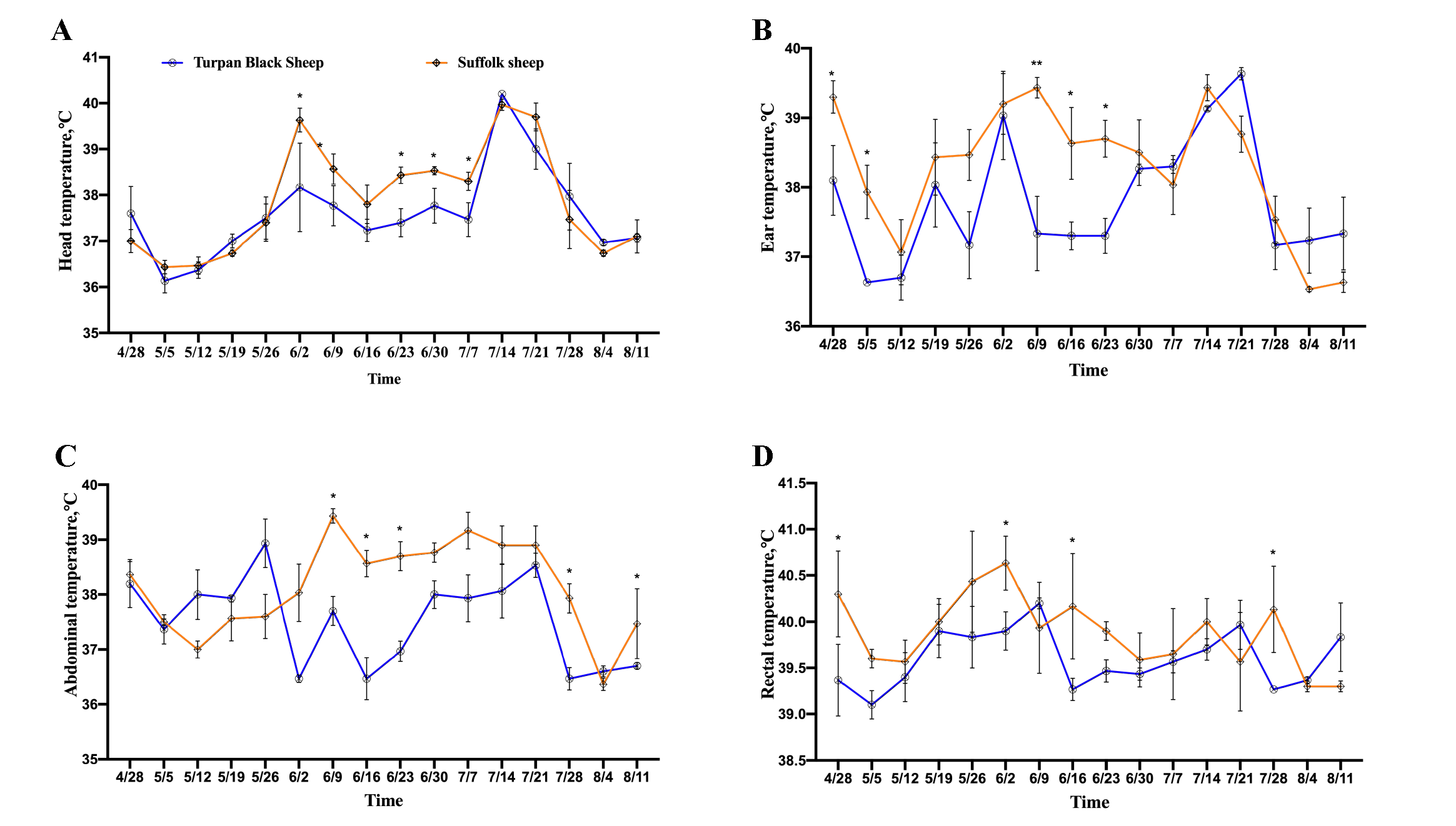

Supplement: Supplementary Figure 1 — Changes in temperature in different parts of the body during heat stress. (A) Head, (B) ear, (C) abdomen, and (D) rectum. [file Image_1.TIF]

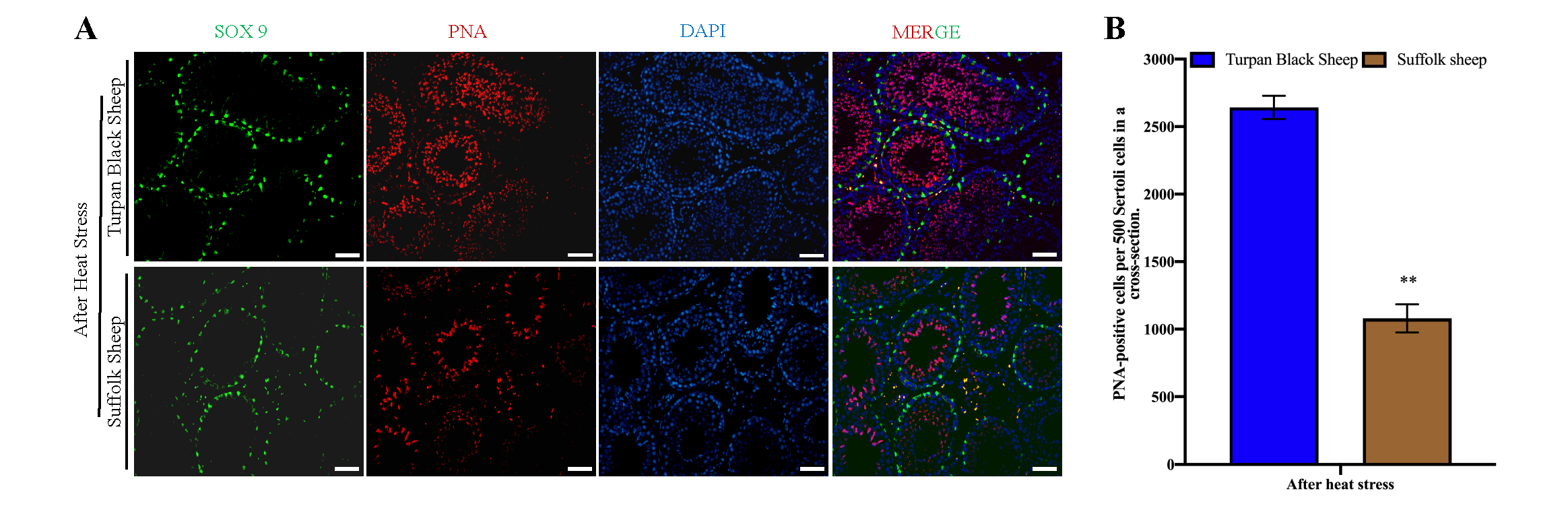

Supplement: Supplementary Figure 2 — Analysis of spermatozoa number in spermatogenic tubules after HS. (A) SOX9 (green fluorescence) labeled supporting cells, PNA (red fluorescence) labeled sperm, and DAPI (blue fluorescence) labeled nucleus. (B) Spermatozoa in 500 sertoli cells. Scale = 50 μm, significance was calculated by T-test, ** means P < 0.01. [file Image_2.TIF]

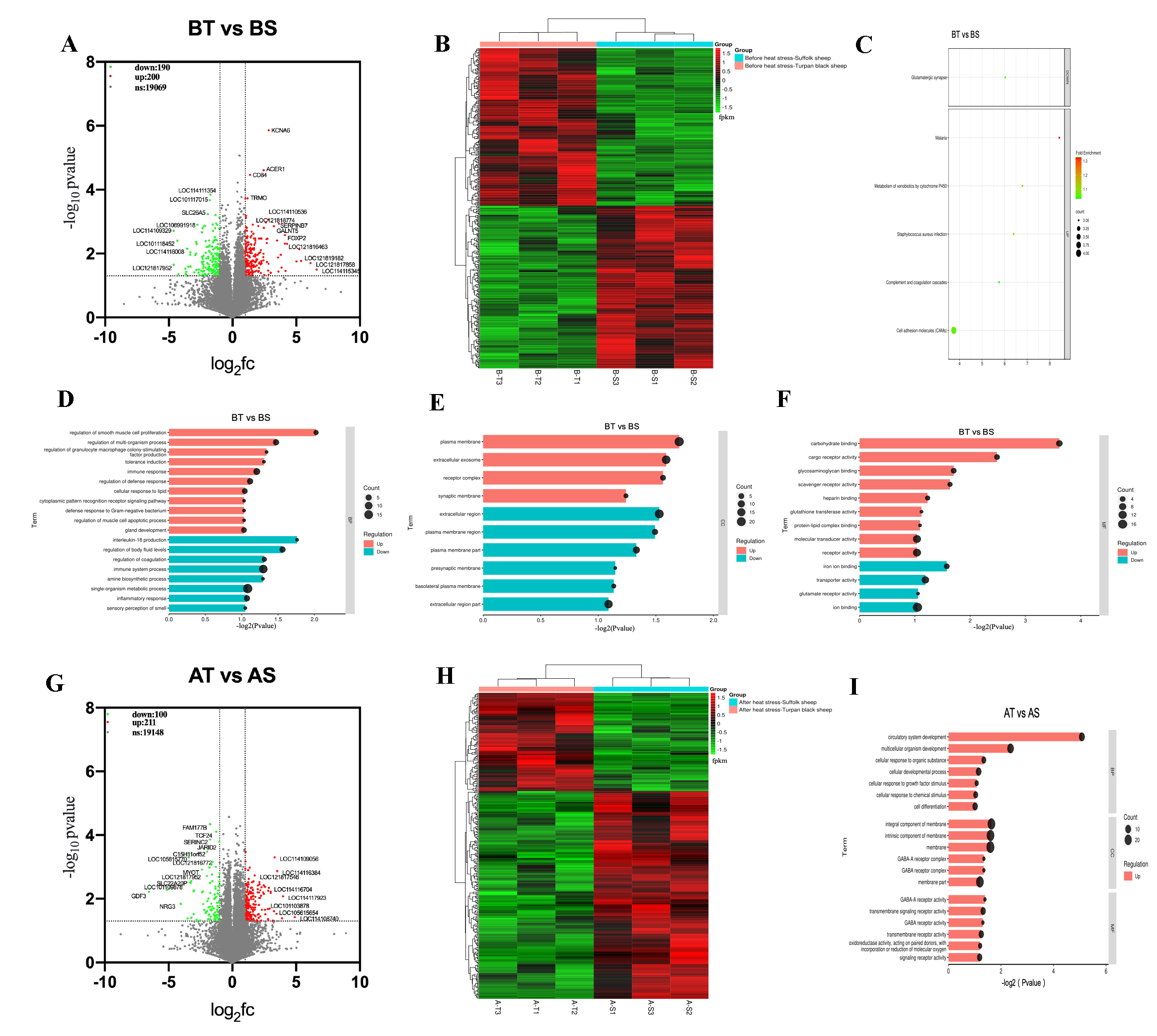

Supplement: Supplementary Figure 3 — Testis transcriptional profile characteristics and differential gene clustering analysis. (A,B) Volcano and heat map of testis transcriptome differential genes in Turpan black sheep and Suffolk sheep before HS. (C–F) KEGG pathway and cluster analysis of the biological process (BP), cellular component (CC), and molecular function (MF) of differential gene enrichment in Turpan black sheep and Suffolk sheep before HS. (G,H) Volcano and heat maps of differentially gene genes in the testicular transcriptomes of Turpan black sheep and Suffolk sheep after HS. (I) Cluster analysis of differential genes BP, CC, and MF in Turpan black sheep and Suffolk sheep after HS. In the volcano plot, red represents up-regulated genes, green represents down-regulated genes, and gray represents genes with no significant difference. The volcano plot showed that the differentially expressed genes satisfy the correction condition P < 0.05. [file Image_3.TIF]
